# Influence of Shenxiong Glucose Injection on the Activities of Six CYP Isozymes and Metabolism of Warfarin in Rats Assessed Using Probe Cocktail and Pharmacokinetic Approaches

**DOI:** 10.3390/molecules22111994

**Published:** 2017-11-20

**Authors:** Jia Sun, Yuan Lu, Yueting Li, Jie Pan, Chunhua Liu, Zipeng Gong, Jing Huang, Jiang Zheng, Lin Zheng, Yongjun Li, Ting Liu, Yonglin Wang

**Affiliations:** 1 Key Laboratory of Pharmaceutics of Guizhou Provincial, Guizhou Medical University, No. 9, Beijing Road, Yunyan District, Guiyang 550004, China; sunjia1202@126.com (J.S.); 18798090340@163.com (Y.L.); nhwslyt@163.com (Y.L.); 18798780192@163.com (J.P.); liu_hua139@126.com (C.L.); gzp4012607@126.com (Z.G.); zhengseattle@gmail.com (J.Z.); mailofzl@126.com (L.Z.); 2 School of Pharmacy, Guizhou Medical University, No.9, Beijing Road, Yunyan District, Guiyang 550004, China; 18111990650@163.com (J.H.); liyongjun026@126.com (Y.L.); 3 National Engineering Research Center of Miao’s Medicines, No. 9, Beijing Road, Yunyan District, Guiyang 550004, China; 4 Engineering Research Center for the Development and Application of Ethnic Medicine and TCM, Ministry of Education, Guizhou Medical University, No.9, Beijing Road, Yunyan District, Guiyang 550004, China

**Keywords:** CYP isozyme, herb-drug interaction, Shenxiong glucose injection, warfarin

## Abstract

Shenxiong glucose injection (SGI), a traditional Chinese medicine (TCM) preparation, has been widely used for the treatment of various cardiovascular and cerebrovascular diseases for many years. We assessed the potential influences of SGI on the activities of six CYP enzymes (CYP1A2, CYP2C11, CYP2C19, CYP2D4, CYP2E1, and CYP3A2) and on the pharmacokinetics of warfarin in rats. We compared plasma pharmacokinetics of six probe drugs (caffeine/CYP1A2, tolbutamide/CYP2C11, omeprazole/CYP2C19, metoprolol/CYP2D4, chlorzoxazone/CYP2E1, and midazolam/CYP3A2) and of warfarin between control and SGI-pretreated groups, to estimate the effect on the relative activities of the six isozymes and warfarin metabolism. There were no significant differences in the pharmacokinetic parameters of caffeine, omeprazole, metoprolol, chlorzoxazone, and midazolam between the SGI-pretreated and control groups. However, many pharmacokinetic parameters of tolbutamide in SGI-pretreated rats were affected significantly (*p* < 0.05), and indicated tolbutamide metabolism in the former group was markedly slower. Moreover, SGI reduced the clearance of warfarin. These results suggested SGI showed no effects on the enzyme activities of rat CYP1A2, CYP2C19, CYP2D4, CYP2E1, and CYP3A2, but inhibited the enzyme activity of CYP2C11, and improved the blood concentration of warfarin. This suggests that the dose of warfarin may need be adjusted when co-administrated with SGI.

## 1. Introduction

Shenxiong glucose injection (SGI) is a preparation containing the water extracts of *Salvia miltiorrhiza* Bunge and ligustrazine hydrochloride. The injection is widely used in China for the treatment of cardiovascular and cerebrovascular diseases, such as angina pectoris [1,2], coronary heart disease [3], myocardial infarction [4,5], acute cerebral infarction [6,7], and ischemic stroke [8]. The randomized controlled trial studies showed that the total efficiency was greater than 79% [8,9,10]. *Salvia miltiorrhiza* Bunge, called red sage or Danshen in Chinese, is one of the most versatile Chinese herbal drugs, and has been used for hundreds of years. It has been suggested that some *Salvia miltiorrhiza* Bunge products may be associated with a number of clinically important herb–drug interactions that can cause adverse outcomes, as well as in interactions with other herbs [11,12,13]. Single-dose and steady-state studies in rats have indicated that Danshen increases the area under the curve (AUC), absorption rate constants, maximum concentrations, and elimination half-lives, but decreases the clearance and apparent volume of distribution of both R- and S-warfarin [14,15,16]. Ligustrazine (2,3,5,6-tetramethylpyrazine) is the main active ingredient of Szechwan lovage rhizome, and has been extensively used to treat cardiovascular and cerebrovascular diseases [17]. However, to date, no systematic study has reported on the impact of SGI on cytochromes P450 enzyme activities.

Warfarin (R- and S-warfarin) has become one of the most widely used medications for the treatment and prevention of thromboembolic events [18]. Although highly effective [19,20,21], warfarin therapy poses risks for hemorrhage (upon overdosing) or ischemic stroke (underdosing), due to its narrow therapeutic window and high inter-individual response variability [20]. Moreover, increasing cases of herb–drug interactions have accumulated, leading to clinical concern [22], moreover, 58 different plants that may alter the blood hemostasis and anticoagulation with warfarin have been reported [23]. Ramsay et al. [24] found that 58% patients who take warfarin in combination with herbal medicines may face the potential to interact with the anticoagulant. Generally, warfarin, clinically available as racemic mixture, is cleared by different metabolic pathways. S-Warfarin is mainly metabolized by CYP2C9 [25], while CYP2C19 [26] and CYP3A (CY4/5/7) [27,28,29] may contribute to minor pathways for metabolism of this enantiomer. Although not studied as extensively, R-warfarin is metabolized mainly by CYPs1A2, 2C19, and 3A, while CYP2C9 contributes to minor metabolic pathways [29,30]. It is noteworthy that P-450 isozymes involved in the metabolism of warfarin include 2C9, 2C19, 2C8, 2C18, 1A2, and 3A4, and 2C9 is likely to be the principal form of human liver P-450 which modulates the in vivo anticoagulant activity of warfarin [31,32]. Therefore, it is important to define how SGI influences the activity of CYP enzymes, and to determine whether SGI modifies the pharmacokinetic behavior of warfarin.

The aims of the present study were to evaluate the effects of SGI on the activities of six rat CYP enzymes (CYPs1A2, 2C11, 2C19, 2D4, 2E1, and 3A2), and to investigate the impact of SGI on the metabolism of warfarin for the safe use of warfarin.

## 2. Results

### 2.1. Effect of SGI on CYP1A2 in Rats

The effects of SGI in the different treatment groups on pharmacokinetic parameters (AUC(0–*t*), AUC(0–∞), MRT(0–*t*), MRT(0–∞), *t*_1/2_, CL, and Vd) of caffeine in rats are presented in Table 1. Little changes in pharmacokinetic parameters of caffeine were observed in rats pretreated with either SGI or vehicle. This indicates that SGI had no influence on CYP1A2 activity in vivo.

### 2.2. Effect of SGI on CYP2C11 in Rats

**Figure 1:**
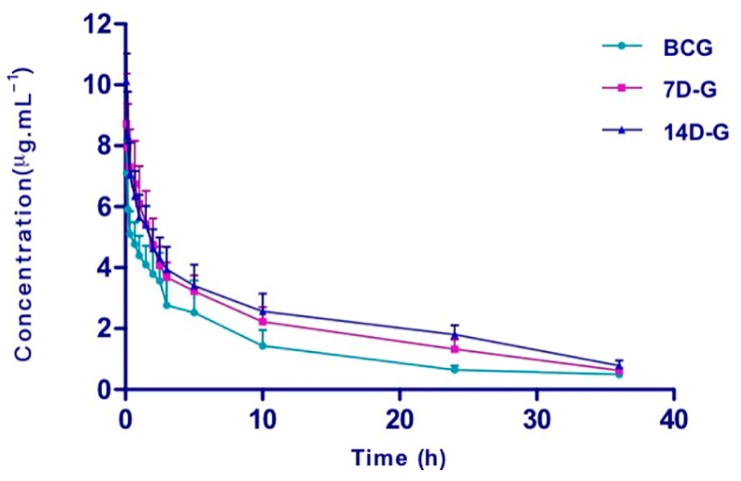
The mean plasma concentration-time curves of tolbutamide (1.0 mg/kg, i.v.) in the different groups (*n* = 6). BCG, blank control group (0.9% sodium chloride solution for 10 days); 7D-G, short-period group (5 mL/kg of SGI concentrated solution, i.v., once daily for consecutive 7 days); 14D-G, long-period group (5 mL/kg of SGI concentrated solution, i.v., once daily for consecutive 14 days). Error bars represent SD.

CYP2C11 activity was evaluated by assessment of tolbutamide pharmacokinetic behavior (Table 2). The mean plasma concentration–time curves of tolbutamide (Figure 1) in the indicated study groups are presented. Pretreatment with SGI caused significant increases in AUC(0–*t*), AUC(0–∞), MRT(0–*t*), MRT(0–∞), and CL of tolbutamide, compared with those observed in the BCG. In addition, significantly increased *t*_1/2_ was observed in 14D-G group pre-treated with SGI. Taken together, these results indicate that SGI may inhibit CYP2C11 activity in vivo.

### 2.3. Effect of SGI on CYP2C19 in Rats

Pharmacokinetic parameters AUC(0–*t*), AUC(0–∞), MRT(0–*t*), MRT(0–∞), t1/2, CL, and Vd of omeprazole in rats are listed in Table 3. There was no significant difference in the pharmacokinetic parameters in the animals between the treatment with SGI and vehicle, indicating that SGI showed little impact on CYP2C19 activity in vivo.

### 2.4. Effect of SGI on CYP2D4 in Rats

Change in CYP2D4 activity induced by SGI was assessed by comparing the pharmacokinetics of metoprolol in rats pretreated with SGI or vehicle, as shown in Table 4. No significant difference in the pharmacokinetic parameters were observed in animals given either SGI or vehicle. These results imply that SGI revealed little influence on CYP2D4 activity in vivo.

### 2.5. Effect of SGI on CYP2E1 in Rats

Pharmacokinetic profiles of chlorzoxazone after SGI treatment were used to describe the activity of CYP2E1. As shown in Table 5, pharmacokinetic parameters AUC(0–*t*), AUC(0–∞), MRT(0–*t*), MRT(0–∞), t1/2, CL, and Vd of chlorzoxazone in rats pretreated with SGI were found to be similar with those of control animals. This indicates that SGI showed no influence on CYP2E1 activity in vivo.

### 2.6. Effect of SGI on CYP3A2 in Rats

Alternation of CYP3A2 activity by SGI was evaluated by monitoring the pharmacokinetics of midazolam, as shown in Table 6. No significant change in pharmacokinetic behavior of midazolam was observed in rats after pretreatment with SGI. This suggests that SGI exhibited no effect on CYP3A2 activity in vivo.

### 2.7. Effect of SGI on the Pharmacokinetics of Warfarin

**Figure 2:**
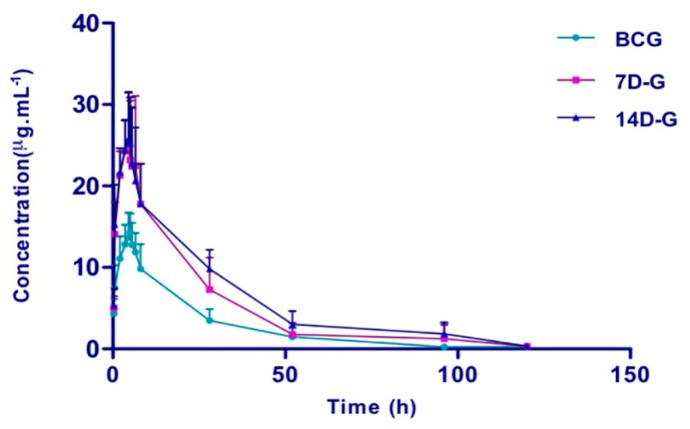
The mean plasma concentration-time curves of warfarin (2.0 mg/kg, i.g.) in the different groups (*n* = 6). BCG, blank control group (0.9% sodium chloride solution for 10 days); 7D-G, short-period group (5 mL/kg of SGI concentrated solution, iv., once daily for consecutive 7 days); 14D-G, long-period group (5 mL/kg of SGI concentrated solution, iv, once daily for consecutive 14 days). Error bars represent SD.

To determine the interaction of SGI with warfarin, time-course plasma warfarin was monitored in rats with and without pretreatment with SGI. The mean plasma concentration–time curves are presented in Figure 2, and the pharmacokinetic parameters are summarized in Table 7. Pretreatment with SGI for 7 days in rats increased the AUC(0–*t*), AUC(0–∞), and C_max_ of warfarin by 86.0%, 87.3%, and 81.4% (all *P* < 0.01), along with decreased CL/F and V/F by 42.9% and 41.2% (both *p* < 0.01), respectively. As expected, more increased AUC(0–*t*), AUC(0–∞), MRT(0–∞), and C_max_ (122.7%, 125.9%, 34.5%, and 83.2%) and more decreased CL/F and V/F (57.1% and 52.9%) were observed in rats pretreated with SGI for 14 days.

## 3. Discussion

Cardiovascular and cerebrovascular diseases that increase the likelihood of thromboembolic events usually require a chronic use of warfarin. In China, warfarin is often used in combination with traditional Chinese medicine (TCM) preparations with function of activating circulation to remove blood stasis, antiplatelet drugs, and non-steroidal anti-inflammatory agents. Prescription analysis showed that the rate of concomitant use of warfarin and TCM, such as SGI, Danshen ligustrazine injection, and xueshuantong injection, was over 50% [33,34]. According scientific research studies, the prothrombin time was prolonged after combined with warfarin and *Salvia miltiorrhiza* Bunge [34]. Recently, there was a report about serious bleeding resulting from warfarin in a patient, which might be caused by co-administration of SGI [35]. This drew our attention to SGI-mediated pharmacokinetic behavior alteration of warfarin, especially the inhibitory effects of SGI on CYP enzymes.

In this study, we chose rats as the experimental animal. Although humans differ from rats with regard to isoform composition, expression, and catalytic activities of drug-metabolizing enzymes, rats are common animal models for metabolic behavior studies [36]. Human CYP2D6 is homolog to rat CYP2D4, human CYP3A4 is homolog to rat CYP3A2, human CYP2C9 is homolog to rat CYP2C11, human CYP1A2, CYP2C19, and CYP2E1 are homologs to rat CYP1A2, CYP2C19, and CYP2E1 respectively. We investigated the potential influence of SGI on the enzyme activities of CYPs1A2, 2C11, 2C19, 2D4, 2E1, and 3A2 in rats, by examining the pharmacokinetic behaviors of their probe substrates—caffeine, tolbutamide, omeprazole, metoprolol, chlorzoxazone, and midazolam, respectively.

Water-soluble extracts of *Salvia miltiorrhiza* were reported to scavenge peroxides and to inhibit the expression of adhesion molecules in vascular endothelium and leukocytes [37]. Recent studies demonstrated that salvianolic acid B and tanshinol, found in water *Salvia miltiorrhiza* extracts showed weak inhibitory effects on CYP enzymes [38,39,40]. However, tanshinones, lipophilic components of *Salvia miltiorrhiza*, revealed considerable inhibition towards CYP1A2, 2C9 and 3A4 [41,42]. SGI is composed of the water extracts of *Salvia miltiorrhiza* and tetramethylpyrazine hydrochloride. The water extracts of *Salvia miltiorrhiza* contain tanshinol, protocatechuic aldehyde, rosmarinic acid, salvianolic acid B, and salvianolic acid A as major components, specifically, 100 mL of SGI contains 20 mg tanshinol and 100 mg tetramethylpyrazine hydrochloride [43]. No information is available for the effect of tetramethylpyrazine hydrochloride on CYP450 activity in vivo. Whether the observed decrease in CYP2C11 activity by SGI resulted from tetramethylpyrazine hydrochloride, which SGI contained, needs further investigation.

Warfarin (R- and S-) is metabolized to 4-hydroxy- and 10-hydroxy-warfarin via CYP3A4; to 6-hydroxy- and 8-hydroxy-warfarin by CYP1A2; to 6-hydroxy- and 7-hydroxywarfarin, through CYP2C9 (in humans) or CYP2C11 (in rats) [30]. The present study demonstrated that co-administration of SGI caused inhibition of CYP2C11, and not of the other CYPs evaluated. It is likely that the observed enhancement of blood concentration of warfarin in rats co-treated with SGI may arise from the inhibition of CYP2C11 activity. Therefore, co-administration of SGI and warfarin may result in herb–drug interactions, which may be an important factor which leads to serious adverse reactions. Further study is needed to evaluate the pharmacodynamic changes and toxicity of warfarin induced by co-administration of SGI.

Furthermore, there is an intrinsic factor to affect warfarin pharmacokinetics (PK) and pharmacodynamics (PD), which is mainly the genetic polymorphisms influencing the expression of CYP2C9 and vitamin K epoxide reductase complex subunit 1 (VKORC1), respectively [44]. Importantly, CYP2C9 and VKORC1 are significantly different among Asians, Europeans, and Africans [45,46], and the Chinese populations are more sensitive to warfarin than Western populations [46,47]. For the genetic polymorphisms, the warfarin label was updated in 2010, and gave a recommendation for initial dosing ranges for patients with different combinations of CYP2C9 and VKORC1 genotypes [48]. Regular monitoring of INR (International Normalized Radio) should be performed on all treated patients.

## 4. Materials and Methods

### 4.1. Chemical and Reagents

Shenxiong glucose injection (Product No. 20131125) and SGI concentrated solution (Product No. 20131103) were obtained from Guizhou Jing Feng injection Co., Ltd. (Guiyang, China). Warfarin tablets (Product No. 201311) were purchased from Shanghai Xinyi Pharmaceutical Co., Ltd. (Shanghai, China). Caffeine (Product No. 12003) was obtained from the China National Institute of Metrology, and puerarin (Product No. 0752-9605) as internal standard (IS) 1, was acquired from China National Institutes for Food and Drug Control. Omeprazole (Product No. 20130408), tolbutamide (Product No. 20120910), chlorzoxazone (Product No. 20121115), metoprolol tartrate (Product No. 56392-17-7), warfarin sodium (Product No. D1206AS), and naproxen sodium (Product No. S0322AS) as IS 2 were obtained from Dalian Meilun Biology Technology Co., Ltd. (Liaoning, China). Midazolam (Product No. 20081213) was obtained from Jiangsu Nhwa Pharmaceutical Co., Ltd. (Jiangsu, China). HPLC-grade acetonitrile and formic acid were supplied by Merck Company Inc. (Darmstadt, Germany). Distilled water was obtained from Watsons Group Co., Ltd. (Hong Kong). All other reagents were of analytical grade and were obtained from Kermel Technology Co., Ltd. (Tianjin, China).

### 4.2. Animals and Treatment

Male Sprague Dawley rats (200 ± 20 g) were purchased from the Laboratory Animal Center of Guizhou Medical University. All animal maintenance and experimental studies were based on the guidelines of the National Institutes of Health for the Care and Use of Animals, and were approved by the Experiment Animal Center of Guizhou Medical University. All rats were maintained in a room under controlled temperature and humidity and fed standard laboratory food and water.

The experimental animals were divided into CYP probe group and warfarin group. The herb/drug administration was designed as follows: the CYP probe group comprised three subgroups (*n* = 6 per subgroup), viz., the blank control group (BCG), short-period group (7D-G), and long-period group (14D-G). BCG received 0.9% sodium chloride solution for 10 days. The other two groups were administered with concentrated SGI solution at a dose of 5.0 mL/kg via caudal vein daily for 7 or 14 consecutive days, respectively. Warfarin group also comprised three sub-groups: the blank control group (BCG), short-period group (7D-G), and long-period group (14D-G). BCG received 0.9% sodium chloride solution for 10 days. The other two groups were treated with concentrated SGI solution at a dose of 5.0 mL/kg via caudal vein daily for 7 or 14 days.

On either the 8th or 15th day, the animals of the CYP probe group were treated with probe cocktail solution (5.0 mL/kg) containing caffeine (1.0 mg/kg), tolbutamide (1.0 mg/kg), omeprazole (2.0 mg/kg), metoprolol (10 mg/kg), chlorzoxazone (4.0 mg/kg), or midazolam (4.0 mg/kg), via caudal vein. Similarly, on either the 8th or 15th day, the rats of the warfarin groups were given warfarin (2.0 mg/kg, i.g.) suspended in CMC-Na solution (5.0 g/L).

The doses selected for the animal study were based on the clinical dose of SGI, which is 100–200 mL as a single daily dose. Thus, the doses used for rats needed to be 10.8–21.7 mL/kg body weight, as calculated for conversion between species. However, the required injection volume would then be in excess of the regular injection volume for rats (10 mL/kg body weight). Therefore, a concentrated form of SGI was used (equivalent to 80 mg per 100 mL of salvianic acid and 400 mg per 100 mL of ligustrazine hydrochloride); this concentration was four times higher than that of the original SGI solution (equivalent to 20 mg per 100 mL of salvianic acid and 100 mg per 100 mL of ligustrazine hydrochloride). To mimic the clinical use of SGI, the effects of SGI on rats CYP enzymes were evaluated after the rats were intravenously administered with SGI for 7 (short period) or 14 consecutive days (long period).

### 4.3. Sample Preparation

#### 4.3.1. CYP Probe–Drug Groups

Blood samples were collected in Eppendorf tubes coated with heparin sodium from rats at 0.083, 0.167, 0.333, 0.667, 1, 1.5, 2, 2.5, 3, 5, 10, 12, 24, and 36 h after dosing, and were immediately centrifuged at 2300 × *g* at room temperature for 5 min. These samples were then stored at −20 °C until used.

All samples (50 µL) spiked with internal standard puerarin (final concentration: 4.0 µg/mL) were mixed with 400 µL of methanol. The resulting mixture was vortexed for 5 min and centrifuged at 20,000 × *g* for 10 min. The supernatants were transferred to a fresh tube and concentrated under nitrogen at 45 °C. The residues were reconstituted with 400 µL of the initial mobile phase for UPLC, followed by vortexing for 1 min and centrifugation at 20,000 × *g* for 5 min.

#### 4.3.2. Warfarin Groups

Blood samples were collected from rats at 0.33, 0.5, 2, 3.5, 4.5, 5, 5.5, 6.5, 8, 28, 54, 96, and 120 h after administration, and were immediately centrifuged at 2300 × *g* at room temperature for 5 min. These samples were also stored at −20 °C until analyzed.

To 50 µL of rat plasma samples, IS 2 naproxen (50 µL, 20.0 µg/mL) and dilute hydrochloric acid (50 µL, 1 M) were added, prior to mixing with 350 µL of methanol. The rest of sample preparation protocols were similar as described for that of CYP probe–drug groups.

### 4.4. Analytical Methods

#### 4.4.1. CYP Probe Groups

UPLC-MS analyses were carried out on a system consisting of a Waters ACQUITYTM UPLC (Waters, Milford, MA, USA) coupled to a Waters ACQUITY triple quadrupole mass spectrometer equipped with a Z-spray ESI source, which was operated in either positive-ion or negative-ion mode detection. Nitrogen was used as a nebulizing gas with a source temperature of 120 °C. Desolvation gas (nitrogen) was heated to 350 °C, and delivered at a flow rate of 650 L/h. Selected or single-ion recording (SIR) was chosen for quantification of the probe substrates (Table 8). The operation of the UPLC-MS and data analysis was achieved using a MassLynxTM V4.1 workstation (Micromass, Manchester, UK).

Liquid chromatography analyses were performed in a gradient elution mode on Waters BEH C18 column (2.1 mm × 50 mm, 1.7 µm) (Waters, Milford, MA, USA) at 45 °C. The mobile phase consisted of 0.1% formic acid in acetonitrile (A) and 0.1% formic acid in water (B). A linear gradient at a flow rate of 0.35 mL/min was run at 10–65% A over 0–3.0 min, 65–90% A over 3.0–3.5 min, 90–10% A over 3.5–4.0 min, and 10% A over 4.0–4.5 min. The samples were maintained at 4 °C CV in the auto-sampler, and a volume of 1 µL was injected into the UPLC system.

#### 4.4.2. Warfarin

The mass spectrometer was operated in positive ion mode. The cone voltage for warfarin and naproxen (IS 2) was set at 40 and 30 V, along with the collision energy at 25 and 15 V for warfarin and naproxen, respectively. Argon was used as collision gas at a flow rate of 0.16 mL/min. Multiple reaction monitoring (MRM) was employed to perform mass spectrometric quantification. The MRM analysis was conducted by monitoring the precursor ion to product ion transitions of *m*/*z* 307.0→164.0 for warfarin and *m*/*z* 230.9→185.0 for naproxen.

The mobile phase consisted of 0.1% formic acid in acetonitrile (A) and 0.1% formic acid in water (B). A linear gradient at a flow rate of 0.35 mL/min was run at 5–30% A over 0–1.0 min, 30–60% A over 1.0–2.0 min, 60–90% A over 2.0–3.0 min, and 5% A over 3.0–4.0 min.

All other analysis conditions were the same as described for that of CYP probe–drug groups.

### 4.5. Statistical Analysis

Data were presented as mean ± SD. Pharmacokinetic parameter calculations were carried out using the DAS 2.0 pharmacokinetic program (Chinese Pharmacological Society, Beijing China), and generated using a non-compartmental model (statistical moment). Statistically significant differences in the pharmacokinetic parameters between the treatment groups and the blank control group were assessed using one-way analysis of variance followed by Dunnett’s test, with the level of statistical significance set at 0.05.

## 5. Conclusions

SGI demonstrated inhibitory effect on CYP2C11 in rats. Co-administration of SGI induced elevation in AUC and C_max_ of warfarin, possibly resulting from inhibition of CYP2C11 activity. This suggests that it is potentially risky when SGI is co-administered with warfarin. Dose adjustment of warfarin may be required when co-administration with SGI is necessary.

## Figures and Tables

**Table 1:** Effects of Shenxiong glucose injection (SGI) on pharmacokinetic parameter of caffeine (1.0 mg/kg).

Parameters	BCG	Treatment Groups	
		7D-G	14D-G
AUC_(0–*t*)_(mg/L·h)	2.62 ± 0.68	2.61 ± 1.09	2.38 ± 0.41
AUC_(0–∞)_(mg/L·h)	3.42 ± 0.79	3.24 ± 0.96	2.92 ± 0.59
MRT_(0–*t*)_(h)	1.37 ± 0.15	1.46 ± 0.18	1.35 ± 0.17
MRT_(0–∞)_(h)	3.70 ± 2.96	3.37 ± 2.01	2.77 ± 1.17
*t*_1/2_(h)	3.33 ± 2.56	2.89 ± 1.84	2.64 ± 1.04
CL(L/h/kg)	0.77 ± 0.18	0.83 ± 0.23	0.89 ± 0.18
Vd(L/kg)	3.40 ± 1.97	3.51 ± 2.24	3.25 ± 1.00

BCG, blank control group (0.9% sodium chloride solution for 10 days); 7D-G, short-period group (5 mL/kg of SGI concentrated solution, iv, once daily for consecutive 7 days); 14D-G, long-period group (5 mL/kg of SGI concentrated solution, iv, once daily for consecutive 14 days). Values are represented as mean ± SD (*n* = 6).

**Table 2:** Effects of SGI on pharmacokinetic parameter of tolbutamide (1.0 mg/kg).

Parameters	BCG	Treatment Groups	
		7D-G	14D-G
AUC_(0–*t*)_(mg/L·h)	47.76 ± 15.23	73.87 ± 12.25 **	85.10 ± 13.42 **
AUC_(0–∞)_(mg/L·h)	53.39 ± 16.71	85.99 ± 13.26 **	106.40 ± 9.93 **
MRT_(0–*t*)_(h)	9.13 ± 1.55	11.52 ± 0.592 **	12.57 ± 0.35 **
MRT_(0–∞)_(h)	13.28 ± 3.71	17.68 ± 1.39 **	22.44 ± 4.44 **
t_1/2_(h)	9.77 ± 3.53	13.00 ± 0.87	16.43 ± 3.35 **
CL(L/h/kg)	0.02 ± 0.008	0.01 ± 0.002 **	0.01 ± 0.001 **
Vd(L/kg)	0.28 ± 0.14	0.22 ± 0.04	0.23 ± 0.06

BCG, blank control group (0.9% sodium chloride solution for 10 days); 7D-G, short-period group (5 mL/kg of SGI concentrated solution, iv, once daily for consecutive 7 days); 14D-G, long-period group (5 mL/kg of SGI concentrated solution, iv, once daily for consecutive 14 days). Values are represented as mean ± SD (*n* = 6). * *p* < 0.05 when compared with related parameters of model rats. ** *p* < 0.01 when compared with related parameters of model rats.

**Table 3:** Effects of SGI on pharmacokinetic parameter of omeprazole (2.0 mg/kg).

Parameters	BCG	Treatment Groups	
		7D-G	14D-G
AUC_(0–*t*)_(mg/L·h)	4.54 ± 0.62	5.26 ± 2.25	5.10 ± 0.88
AUC_(0–∞)_(mg/L·h)	6.10 ± 0.96	7.00 ± 2.42	6.44 ± 1.19
MRT_(0–*t*)_(h)	0.90 ± 0.06	0.95 ± 0.15	0.82 ± 0.10
MRT_(0–∞)_(h)	2.15 ± 0.37	2.39 ± 1.06	1.82 ± 0.45
*t*_1/2_(h)	1.92 ± 0.38	2.04 ± 1.15	1.78 ± 0.47
CL(L/h/kg)	1.68 ± 0.28	1.57 ± 0.50	1.59 ± 0.26
Vd(L/kg)	4.52 ± 0.43	4.67 ± 2.59	4.00 ± 0.71

BCG, blank control group (0.9% sodium chloride solution for 10 days); 7D-G, short-period group (5 mL/kg of SGI concentrated solution, i.v., once daily for consecutive 7 days); 14D-G, long-period group (5 mL/kg of SGI concentrated solution, iv, once daily for consecutive 14 days). Values are represented as mean ± SD (*n* = 6).

**Table 4:** Effects of SGI on pharmacokinetic parameter of metoprolol (10.0 mg/kg).

Parameters	BCG	Treatment Groups	
		7D-G	14D-G
AUC_(0–*t*)_(mg/L·h)	5.02 ± 1.32	5.25 ± 2.73	6.48 ± 1.96
AUC_(0–∞)_(mg/L·h)	6.00 ± 1.51	6.01 ± 3.07	7.85 ± 2.12
MRT_(0–*t*)_(h)	1.71 ± 0.17	1.74 ± 0.08	1.61 ± 0.16
MRT_(0–∞)_(h)	2.72 ± 0.65	2.5 ± 0.41	2.9 ± 0.95
*t*_1/2_(h)	1.92 ± 0.56	1.77 ± 0.51	2.27 ± 1.07
CL(L/h/kg)	1.76 ± 0.45	1.98 ± 0.75	1.36 ± 0.37
Vd(L/kg)	4.76 ± 1.56	4.96 ± 2.05	4.43 ± 2.35

BCG, blank control group (0.9% sodium chloride solution for 10 days); 7D-G, short-period group (5 mL/kg of SGI concentrated solution, iv, once daily for consecutive 7 days); 14D-G, long-period group (5 mL/kg of SGI concentrated solution, iv, once daily for consecutive 14 days). Values are represented as mean ± SD (*n* = 6).

**Table 5:** Effects of SGI on pharmacokinetic parameter of chlorzoxazone (4.0 mg/kg).

Parameters	BCG	Treatment Groups	
		7D-G	14D-G
AUC_(0–*t*)_(mg/L·h)	19.23 ± 2.72	21.49 ± 1.82	21.56 ± 2.21
AUC_(0–∞)_(mg/L·h)	20.45 ± 2.81	23.05 ± 2.54	23.26 ± 2.55
MRT_(0–*t*)_(h)	2.45 ± 0.18	2.50 ± 0.23	2.67 ± 0.33
MRT_(0–∞)_(h)	3.11 ± 0.14	3.28 ± 0.80	3.49 ± 0.62
*t*_1/2_(h)	2.44 ± 0.06	2.68 ± 0.82	2.72 ± 0.39
CL(L/h/kg)	0.20 ± 0.03	0.18 ± 0.02	0.17 ± 0.02
Vd(L/kg)	0.70 ± 0.12	0.67 ± 0.15	0.68 ± 0.12

BCG, blank control group (0.9% sodium chloride solution for 10 days); 7D-G, short-period group (5 mL/kg of SGI concentrated solution, iv, once daily for consecutive 7 days); 14D-G, long-period group (5 mL/kg of SGI concentrated solution, iv, once daily for consecutive 14 days). Values are represented as mean ± SD (*n* = 6).

**Table 6:** Effects of SGI on pharmacokinetic parameter of midazolam (4.0 mg/kg).

Parameters	BCG	Treatment Groups	
		7D-G	14D-G
AUC_(0–*t*)_(mg/L·h)	6.16 ± 0.38	6.17 ± 0.92	6.62 ± 1.59
AUC_(0–∞)_(mg/L·h)	6.25 ± 0.40	6.65 ± 1.46	6.73 ± 1.56
MRT_(0–*t*)_(h)	2.39 ± 0.16	2.36 ± 0.14	2.25 ± 0.34
MRT_(0–∞)_(h)	2.53 ± 0.17	3.15 ± 0.92	2.42 ± 0.43
*t*_1/2_(h)	1.76 ± 0.12	2.55 ± 1.23	1.83 ± 0.38
CL(L/h/kg)	0.64 ± 0.04	0.62 ± 0.13	0.62 ± 0.15
Vd(L/kg)	1.63 ± 0.16	2.12 ± 0.58	1.67 ± 0.55

BCG, blank control group (0.9% sodium chloride solution for 10 days); 7D-G, short-period group (5 mL/kg of SGI concentrated solution, iv, once daily for consecutive 7 days); 14D-G, long-period group (5 mL/kg of SGI concentrated solution, iv, once daily for consecutive 14 days). Values are represented as mean ± SD (*n* = 6).

**Table 7:** Effects of SGI on pharmacokinetic parameter of warfarin (2.0 mg/kg).

Parameters	BCG	Treatment Groups	
		7D-G	14D-G
AUC_(0–*t*)_(mg/L·h)	327.11 ± 90.75	608.50 ± 202.37 **	728.37 ± 66.58 **
AUC_(0–∞)_(mg/L·h)	329.91 ± 90.25	617.06 ± 207.37 **	745.28 ± 65.71 **
MRT_(0–*t*)_(h)	21.51 ± 3.58	22.40 ± 6.87	27.63 ± 5.38
MRT_(0–∞)_(h)	22.70 ± 3.32	24.02 ± 7.28	30.54 ± 6.63 **
*t*_1/2z_(h)	17.71 ± 1.95	19.69 ± 4.83	21.5 ± 5.46
T_max_(h)	4.92 ± 0.20	4.67 ± 1.17	4.42 ± 0.49
CLz/F(L/h/kg)	0.007 ± 0.002	0.004 ± 0.001 **	0.003 ± 0.000 **
Vz/F(L/kg)	0.17 ± 0.07	0.10 ± 0.04 **	0.08 ± 0.02 **
C_max_(mg/L)	14.72 ± 2.45	26.70 ± 6.29 **	26.97 ± 4.47 **

BCG, blank control group (0.9% sodium chloride solution for 10 days); 7D-G, short-period group (5 mL/kg of SGI concentrated solution, iv, once daily for consecutive 7 days); 14D-G, long-period group (5 mL/kg of SGI concentrated solution, iv, once daily for consecutive 14 days). Values are represented as mean ± SD (*n* = 6). * *p* < 0.05 when compared with related parameters of model rats. ** *p* < 0.01 when compared with related parameters of model rats.

**Table 8:** Single-ion recording (SIR) transitions and cone voltages for the detection of CYP probe substrates and internal standard.

Probe Drugs	Polarity	Molecular Mass	Parent (*m*/*z*)	Cone (V)
Caffeine	ESI+	194	195	35
Tolbutamide	ESI–	270	269	40
Omeprazole	ESI+	345	346	35
Metoprolol	ESI+	267	268	20
Chlorzoxazone	ESI–	169	168	35
Midazolam	ESI+	325	326	30
Puerarin (IS 1)	ESI+	416	417	40

## Abbreviations

SGI	Shenxiong glucose injection
TCM	the traditional Chinese medicine
AUC	the area under the curves
MRT	mean retention time
Cmax	maximum concentrations
*t*_1/2_	elimination half-lives
CL	the clearance
V	apparent volume of distribution
BCG	blank control group (0.9% sodium chloride solution for 10 days)
7D-G	short-period group (5 mL/kg of SGI concentrated solution, i.v., once daily for consecutive 7 days)
14D-G	long-period group (5 mL/kg of SGI concentrated solution, i.v., once daily for consecutive 14 days)
